# New Insights on Cytological and Metabolic Features of *Ostreopsis* cf. *ovata* Fukuyo (Dinophyceae): A Multidisciplinary Approach

**DOI:** 10.1371/journal.pone.0057291

**Published:** 2013-02-27

**Authors:** Giorgio Honsell, Alois Bonifacio, Marco De Bortoli, Antonella Penna, Cecilia Battocchi, Patrizia Ciminiello, Carmela Dell’Aversano, Ernesto Fattorusso, Silvio Sosa, Takeshi Yasumoto, Aurelia Tubaro

**Affiliations:** 1 Department of Agricultural and Environmental Sciences, University of Udine, Udine, Italy; 2 Department of Engineering and Architecture, University of Trieste, Trieste, Italy; 3 Department of Life Sciences, University of Trieste, Trieste, Italy; 4 Department of Biomolecular Sciences, Laboratory of Environmental Biology, University of Urbino, Pesaro, Italy; 5 Department of Natural Products Chemistry, University of Naples “Federico II”, Naples, Italy; 6 Japan Food Research Laboratories, Tama Laboratory, Tokyo, Japan; University of Connecticut, United States of America

## Abstract

The harmful dinoflagellate *Ostreopsis* cf. *ovata* has been causing toxic events along the Mediterranean coasts and other temperate and tropical areas, with increasing frequency during the last decade. Despite many studies, important biological features of this species are still poorly known. An integrated study, using different microscopy and molecular techniques, Raman microspectroscopy and high resolution liquid chromatography-mass spectrometry (HR LC-MS), was undertaken to elucidate cytological aspects, and identify main metabolites including toxins. The species was genetically identified as *O.* cf. *ovata*, Atlantic-Mediterranean clade. The ultrastructural results show unique features of the mucilage network abundantly produced by this species to colonize benthic substrates, with a new role of trichocysts, never described before. The amorphous polysaccharidic component of mucilage appears to derive from pusule fibrous material and mucocysts. In all stages of growth, the cells show an abundant production of lipids. Different developmental stages of chloroplasts are found in the peripheral cytoplasm and in the centre of cell. *In vivo* Raman microspectroscopy confirms the presence of the carotenoid peridinin in *O*. cf. *ovata*, and detects in several specimen the abundant presence of unsaturated lipids structurally related to docosahexaenoic acid. The HR LC-MS analysis reveals that ovatoxin-a is the predominant toxin, together with decreasing amounts of ovatoxin-b, -d/e, -c and putative palytoxin. Toxins concentration on a per cell basis increases from exponential to senescent phase. The results suggest that benthic blooms of this species are probably related to features such as the ability to create a unique mucilaginous sheath covering the sea bottom, associated with the production of potent toxins as palytoxin-like compounds. In this way, *O.* cf. *ovata* may be able to rapidly colonize benthic substrates outcompeting other species.

## Introduction

During the last years, the benthic dinoflagellate *Ostreopsis ovata* Fukuyo has been associated with several toxic events in temperate waters [1], [2]. In fact, although the genus *Ostreopsis* was at first found in tropical or subtropical waters, since the end of 1990 s it frequently appeared forming blooms also at mid latitudes [3]. In particular, in the Mediterranean Sea, blooms of *O.* cf. *ovata* have been linked with respiratory problems, rhinorrhoea, cough, fever over 38°C, dermatitis and sometimes neutrophilia, in people exposed to marine aerosols and/or directly to seawater [4], [5], [6]. These events might provoke significant economic losses to the tourism industry along the Spanish, Italian and French Mediterranean coasts (7).

Both sanitary and economic problems related to *O.* cf. *ovata* blooms led to focus on this species to investigate different aspects of its biology and ecology for the management of monitoring programs, as well as to try to forecast its blooms. To this purpose, *O.* cf. *ovata* has been recently studied mainly for its taxonomy and genetic profile [3], [8], [9], [10], toxin content [11], [12], [13], [14], cell physiology and bloom ecology [1], [15], [16], [17], [18], [19]. On the contrary, other important cytological and biochemical features have been scarcely considered so far.

Cell ultrastructure of *Ostreopsis* and other toxic benthic gonyaulacoid dinoflagellates, such as *Gambierdiscus* and *Coolia,* was partially described only in a few not recent studies [20], [21], [22]. Besada et al. [20] outlined that all these species show many typical dinoflagellate features, but reveal the presence of a previously undescribed organelle consisting of an array of vesicles containing fibrous material; they suggested a link between this organelle and the enormous amount of mucilage secreted. Mucilage has a fundamental role in *Ostreopsis* growth strategy to colonize benthic substrates [8], [16], [20], and it has been related to a possible micropredation mechanism [23], [24]. However, despite its importance, the mucilaginous network surrounding cells has not been characterized yet from a cytochemical and ultrastructural point of view.

*O. ovata* is considered a harmful species: the toxicity of *Ostreopsis* blooms is associated with the presence of palytoxin-like compounds in the algal cells. Palytoxin (PLTX) is one of the most potent natural toxins so far known: some human fatalities are ascribed to the ingestion of PLTX contaminated fish and crabs, as well as severe human poisonings in inter-tropical areas [6]. A putative palytoxin (pPLTX) [25] and five new palytoxin-like compounds, named ovatoxin-a (OVTX-a) [26], −b, −c, −d, and −e [11] were recently detected by liquid chromatography-mass spectrometry (LC-MS) in field samples and cultures of *O.* cf. *ovata* from Italian [17], [27], [28] and Croatian coasts [29]. Furthermore, in natural samples of *O.* cf. *ovata* collected in the Gulf of Trieste (Italy), PLTX-like compounds have been localized in the cell cytoplasm by immunocytochemistry [30]. Generally, OVTX-a represents the major component of all *O.* cf. *ovata* toxin profiles determined so far, accounting for up to 89% of the total toxin content, followed by OVTX-b, -d+e, -c and pPLTX (listed in order of decreasing concentration). Very recently, *O.* cf. *ovata* isolates from the Central Adriatic Sea were found to have peculiar toxin profiles: an isolate from Numana was found not to produce OVTX-b and –c [13] and an isolate from Portonovo was dominated by a new ovatoxin, designated OVTX-f, which accounted for 50% of the total toxin content [14].

To increase the knowledge on this harmful algal species, an integrated approach was undertaken to elucidate its ultrastructure and metabolic profile, including toxins. In fact, despite many studies carried out on *O.* cf. *ovata* during the last years, cytological aspects have been nearly always overlooked, considering only cell morphology for taxonomical purposes. Many important ultrastructural features of cells are still poorly known. The aim of this study is to provide new insights on ultrastructural and biochemical features of this species not described before, or only partially known. Different techniques, such as light and fluorescence microscopy, scanning and transmission electron microscopy as well as molecular sequence analysis were applied to a Mediterranean clone of *O.* cf. *ovata*. High resolution (HR) LC-MS was used to define the toxin profile both qualitatively and quantitatively. Furthermore, a new approach based on Raman microspectroscopy [31], [32] was applied to detect and localize primary (lipids and polysaccharides) and some secondary metabolites, such as carotenoids, in cells.

## Materials and Methods

### Ethics Statement

No specific permission was required for sampling of *Ostreopsis* as the location is not privately owned or protected. Moreover, the sampling did not involve endangered or protected species.

### Substances

Palytoxin was purchased from Wako Chemicals (Neuss, Germany); Alcian Blue 8GX, Nile Red and poly-ornithin were purchased from Sigma Aldrich (Milan, Italy); peridinin was kindly provided by prof. Takeshi Yasumoto; fluorite (CaF_2_) microscope slides were purchased from Crystal GmbH (Berlin, Germany). Calcofluor White M2R was purchased from Polysciences Inc. (Warrington, USA). Other substances of analytical grade were purchased from Sigma Aldrich (Milan, Italy).

### Cultures

*O.* cf. *ovata* was isolated from phytoplankton net samples, collected along the rocky beach of Canovella de’ Zoppoli in the Gulf of Trieste (Northern Adriatic Sea, Italy) on 8^th^ October 2008. Cell isolation and culturing conditions were as previously described by Penna et al. [9]. *O.* cf. *ovata* C5 strain was used in different experimental procedures of electron microscopy, Raman microspectroscopy, HR LC-MS toxin content analyses, and genotype sequence analysis. Cultures were grown in 1 L glass bottles containing 0.6 L sterilized f/4 medium [33] at 23±1°C with an initial inoculum of 3.0×10^4^ cells; light was provided by cool white fluorescent bulbs (photon flux of 100 µE m^−2^ s^−1^) with a 14∶10 light/dark cycle. Culture sub-samples were fixed with Lugol’s iodine [34] and counted using the Utermöhl method [35]. Culture subsamples of exponential (day 10 after culture inoculum), stationary (day 18) and senescent (day 25) growth phases were harvested by centrifugation at 4000×*g* for 15 min or left to settle on poly-L-lysine coated coverslips (only for scanning electron microscopy). Cells were carefully washed with sterile artificial seawater and prepared for different experimental procedures as follows: i) for transmission electron microscopy (TEM) cell pellets were fixed in two ways: with 2% glutaraldehyde in 0.1 M cacodylate buffer or with 1% osmium tetroxide dissolved in sterile seawater; ii) for scanning electron microscopy (SEM) cells were fixed with 2% glutaraldehyde in sterile seawater; iii) for Raman analysis, culture sub-samples were fixed with 2% paraformaldehyde directly in culture medium; iv) for chemical HR LC-MS analyses, aliquots of culture sub-samples containing 3.0×10^6^ cells collected at different growth phases were stored at −80°C until analyses.

### Molecular Analyses

Genomic DNA was extracted from 10 mL cultures in logarithmic growth phase using the DNeasy Plant Kit (Qiagen, Valencia, CA, USA), according to the manufacturer’s instructions. Polymerase chain reaction (PCR) amplification of 5.8S - ITS ribosomal gene has been described in Penna et al. [36]. The cloning of amplified PCR fragments and sequencing were carried out as in Penna et al. [36]. Alignment of nucleotide sequence against all *Ostreopsis* spp. sequences was made using BLAST with default settings. The sequence of C5 strain was deposited in EMBL (European Molecular Biology Laboratory) and the sequence accession number is JX065591.

### Light Microscopy

Observations were carried out on unfixed and fixed samples using a Leitz Diavert inverted microscope (Ernst Leitz Wetzlar GmbH; Wetzlar, Germany) using bright field, phase contrast and differential interference contrast (DIC) illumination. Cells fixed in 1.25% glutaraldehyde were stained with a 0.02% aqueous solution of Alcian Blue 8GX in 0.06% acetic acid (pH 2.5) to highlight both sulphated and carboxylated extracellular polysaccharides [37].

### Epifluorescence Microscopy

Cells were observed using a Leitz Fluovert inverted microscope (Ernst Leitz Wetzlar GmbH; Wetzlar, Germany) at 400x. Observations to detect chloroplast autofluorescence were carried out using two different filter blocks (I2/3: exciter filter BP 450–490, dichroic beam splitting mirror RKP 510, barrier filter LP 515; N2.1: exciter filter BP 515–560, dichroic beam splitting mirror RKP 580, barrier filter LP 590). Cells were stained with Calcofluor White M2R and observed with UV excitation filter block A (exciter filter BP 340–380, dichroic beam splitting mirror RKP 400, barrier filter LP 425) to show thecal plates [38].

Cells were stained with Nile Red, a selective fluorescent dye for cytoplasmic lipid droplets at final concentration 1∶100 of 1 mg Nile Red/mL acetone stock solution [39] and observed with blue excitation (filter block I2/3) to visualize Nile Red yellow gold fluorescence. Formaldehyde fixed cells were stained with 4′,6-diamidino-2-phenylindole dihydrochloride (DAPI) to highlight nucleus and observed with UV excitation (filter block A).

### Scanning Electron Microscopy

Cultures subsamples were left to settle on poly-L-lysine coated coverslips (BD Biosciences; San Jose, USA), fixed with 2% EM grade glutaraldehyde dissolved in filtered seawater for 30 min, washed in 1∶1 seawater/distilled water and then in distilled water, dehydrated in a gradual series of ethanol, critical point dried with liquid carbon dioxide, sputter coated with gold and observed with a LEICA STEREOSCAN 430i scanning electron microscope (Leica Microsystems; Wetzlar, Germany).

### Transmission Electron Microscopy

Cells in different growth phases were harvested by centrifugation at 4000×*g* for 15 min and fixed with 1% osmium tetroxide dissolved in sterile seawater (fixation 1) or with 2% glutaraldehyde in 0.1 M cacodylate buffer followed by 1% osmium tetroxide post-fixation (fixation 2). Cells were then washed three times with distilled water, dehydrated in a gradual series of ethanol, replaced then by propylene oxide, embedded in Epon Araldite and cut by a diamond knife. Sections were stained by uranyl acetate and lead citrate and observed by a PHILIPS EM 208 transmission electron microscope (FEI; Eindhoven, The Netherlands). Images were acquired by a Gatan wide angle CCD camera (Gatan Inc.; Pleasanton, USA). For negative staining a drop of cell suspension was put on a carbon coated grid and fixed with osmium tetroxide vapours for 5 min. Then, it was washed with distilled water and stained with uranyl acetate for 5 min.

### Raman Microspectroscopy

Raman spectra and maps were acquired using an inVia Raman system (Renishaw plc, Wotton-under-Edge, UK), equipped with a ProScanTMII motorized stage (Prior, Cambridge, UK), a 300 mW diode NIR laser emitting at 785 nm (Renishaw), an edge filter for Rayleigh line rejection, a single-grating spectrograph with a 1800 l/mm grating and a Peltier-cooled (−70°C) CCD detector. Spectral resolution, measured as the full-width at half height of the emission lines of a Ne lamp, was 4 cm^−1^.

Sample consisted in dry solid samples of peridinin and palytoxin, and *O.* cf. *ovata* cells placed on a CaF_2_ microscope slide coated with poly-ornithine and immersed in artificial seawater. Poly-ornithine coating was necessary to ensure cell adhesion (cells were allowed to adhere for 1 h) and thus to prevent cells from moving or floating away during data acquisition. *O.* cf. *ovata* cells were investigated: i) alive, ii) fixed with 2% paraformaldehyde (5 min at RT), and iii) depigmented after fixation upon washing 3 times with an 1∶1 acetone:hexane solution for 5 min at 4°C in order to remove carotenoids. After fixation or depigmentation, cells were centrifuged and resuspended in artificial seawater.

A total of 24 living *O.* cf. *ovata* cells were investigated by acquiring a single Raman spectrum from the middle region of each cell, whereas Raman maps were collected for a total 5 fixed cells and 3 fixed and depigmented cells. For all the cells investigated, the laser was focused on the samples by a 60× Nikon Fluor water dipping objective (N.A. 1.00, working distance 2.0 mm), whereas for dry solid samples of peridinin and palytoxin a 50× Leica N Plan (N.A. 0.75, working distance 0.5 mm) was used.

The laser power at the sample was of 80 mW for all measurements but for peridinin, for which a laser power of 0.1 mW was used to avoid sample photodegradation.

Raman maps of varying dimensions (from about 1600 to 6000 spectra) were acquired using the Streamline™ fast imaging configuration and the WiRE 3.1 software (Renishaw), with steps of 1.2 µm and with a total collection time varying from 5 min (for living cells) to 1 h and 45 min (for fixed and depigmented cells).

Lateral resolution in Raman maps was determined by the map step (1.2 µm), since the 60× objective focused the laser in a spot of less than 1 µm diameter. Single spectra from living cells and from peridinin were collected using an exposure time of 60 s, single spectra from palytoxin were collected using an exposure of 10 s.

Raman maps and spectra were then pre-processed (cosmic rays removal and baseline correction) and analysed (calculation of spectra average and standard deviation, PCA, production of univariate and multivariate images based on the Raman maps) with the hyperSpec software package [40] for R [41]. With the exception of the spectra of palytoxin, all spectra were baseline corrected upon subtracting a polynomial baseline of 2-nd order, to eliminate the sloping background due to fluorescence [42], [43]. Before PCA analysis and averaging, spectral intensity was vector-normalized.

### High Resolution Liquid Chromatography-Mass Spectrometry (HR LC-MS)

Cell pellets of Adriatic *O.* cf. *ovata* cultures were collected by gravity filtration at the end of the following growth phases: exponential (N° cells = 3,251,598) stationary (N° cells = 5,480,526) and senescent (N° cells = 3,083,840). Each pellet sample was added of 3 mL methanol/water (1∶1, v/v) and sonicated for 30 min in pulse mode, while cooling in ice bath. The mixture was centrifuged at 3000 x *g* for 30 min, the supernatant was decanted and the pellet was washed twice with 3 mL of methanol/water (1∶1, v/v). The extracts were combined to a final volume of 9 mL. The obtained mixture was analysed directly by HR LC-MS (5 µL injected) in comparison to palytoxin standard and an *O.* cf. *ovata* extract previously analysed which contained all the ovatoxins so far known [14]. Recovery percentage of the above extraction procedure (98%) was used to correct quantitative results of HR LC-MS analyses.

HR LC-MS experiments were carried out on a hybrid linear ion trap LTQ Orbitrap XL™ Fourier Transform MS (FTMS) equipped with an ESI ION MAX™ source (Thermo-Fisher; San Josè, CA, USA) coupled to an Agilent 1100 LC binary system (Palo Alto, CA, USA). The following LC conditions were used: 3 µm gemini C18 (150×2.00 mm) column (Phenomenex; Torrance, CA, USA); mobile phase: A = water, 30 mM acetic acid, B = 95% acetonitrile/water, 30 mM acetic acid; gradient: 20–100% B over 10 min. and hold 4 min; flow rate: 0.2 mL/min. HR full MS experiments (positive ions) were acquired in the range *m/z* 800–1400 at a resolving power of 15,000. The following source settings were used: spray voltage = 4 kV, capillary temperature = 290°C, capillary voltage = 22 V, sheath gas = 35, auxiliary gas = 1 (arbitrary units), tube lens voltage = 110 V. HR collision induced dissociation (CID) LC-MS^2^ experiments were carried out for confirming identity of individual toxins as reported previously [11], [14].

In quantitative studies, the calibration curve of palytoxin standard (triplicate injection) at four levels of concentration (25, 12.5, 6.25, and 3.13 ng mL^−1^) was used and ovatoxins’ molar responses were assumed to be similar to palytoxin’s. Extracted ion chromatograms (XIC) were obtained by selecting the most abundant ion peaks of both [M+2H-H_2_O]^2+^ and [M+H+Ca]^3+^ ion clusters contained in HR full MS spectra of palytoxin and ovatoxins [11], [44]. A mass tolerance of 5 ppm was used.

## Results

### Molecular Analysis of Genotype Identification

The final alignment of *Ostreopsis* C5 strain sequence of 5.8-ITS rDNA with all *Ostreopsis* spp. sequences from GenBank gave 100% of identity with *O.* cf. *ovata* ribosomal sequence.

### Light and Epifluorescence Microscopy

**Figure 1:**
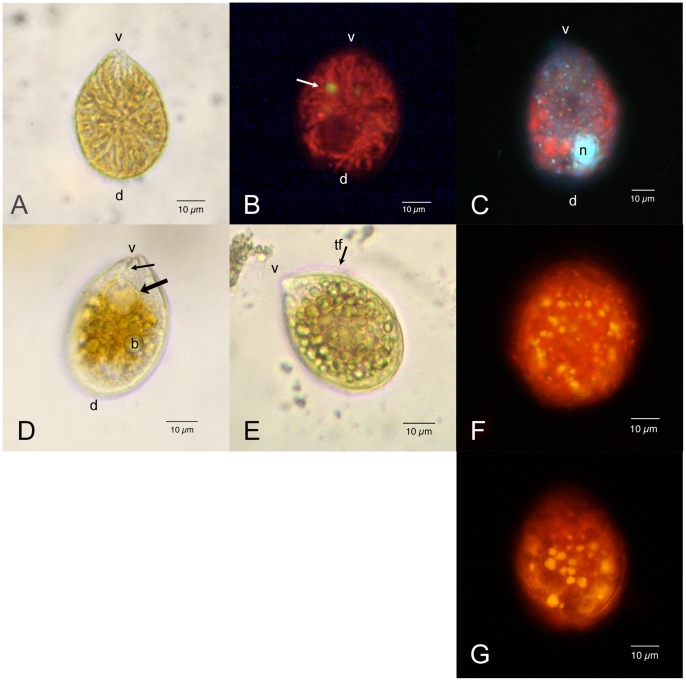
*Ostreopsis* cf. O*vata:* Light and epifluorescence microscopy. Scale bars represent 10 µm. (A) Living cell: bright field microscopy. The cell body is anterio-posteriorly compressed and shows a typical oval tear-shaped morphology pointed to the ventral side (v). The dorsal side (d) is rounded. Numerous elongated yellow brownish chloroplasts radiate from the centre of the cell. (B) Living cell viewed by epifluorescence microscopy under blue excitation (exciter filter BP 450–490, barrier filter LP 515): chloroplasts show an intense red autofluorescence; a small rounded body with yellow autofluorescence (arrow) is also visible. The dark non fluorescent area in the dorsal part of the cell is occupied by the nucleus. (C) Formaldehyde fixed cell stained with DAPI viewed by epifluorescence microscopy under UV excitation (exciter filter BP 340–380, barrier filter LP 425). The nucleus (n) occupies the dorsal part of the cell. Numerous small fluorescent dots (probably chloroplast DNA) are visible in correspondence with chloroplasts, which show a weak red autofluorescence. (D) Living cell: bright field microscopy. It can be observed the pusule (arrow), connected by a narrow canal (small arrow) to the cell ventral end (v). Some rounded translucent bodies (b) are evident in the cytoplasm. (E) Living cell: bright field microscopy. Most cytoplasm appears to be occupied by many rounded translucent bodies, which obscure other cell structures. It is visible the transverse flagellum (tf) running around the cell in the girdle. (F) Exponentially growing cell stained with Nile Red viewed by epifluorescence microscopy under blue excitation. Numerous yellow fluorescent lipid droplets are present in the peripheral cytoplasm. (G) Stationary phase cell stained with Nile Red. Yellow fluorescent lipid droplets appear to be larger.

*O.* cf. *ovata* cells (Figs. 1A–1E) are anterio-posteriorly compressed and show a typical oval tear-shaped morphology pointed to the ventral side. They present many yellow brownish elongated chloroplasts, radiating from the centre of the cell (Fig. 1A). Chloroplasts, when observed by epifluorescence microscopy, show an intense red autofluorescence under blue (Fig. 1B) and also green excitation. This fact is likely to be related to the abundant presence of accessory photosynthetic pigments, such as carotenoids, in addition to chlorophylls. Few small yellow fluorescing rounded bodies are visible in the inner part of the cell under blue light excitation (Fig. 1B, arrow).

The nucleus, made visible by DAPI staining, has a rounded shape (diameter of about 9–12 µm) and occupies the dorsal part of the cell. DAPI staining highlights also small fluorescent dots in correspondence with chloroplasts detectable by their red autofluorescence (Fig. 1C). The ventral side of the cell is pointed and appears always transparent with small granulations; in some cells a large rounded transparent vacuole-like chamber connected by a narrow canal to the ventral end of the cell can be observed (Fig. 1D, arrows).

Thecal plates appear smooth and covered by small pores sparsely scattered over their surface, when observed by epifluorescence microscopy after staining with Calcofluor White M2R.

In wild and cultured cells, cytoplasm is often full of small rounded translucent bodies that obscure cell organelles and do not allow a clear view of nucleus, chloroplasts, and other cell structures (Fig. 1E). These bodies, stained by the lipid specific fluorochrome Nile Red, show an intense yellow fluorescence, revealing they are neutral lipid droplets (Figs 1F, 1G). Their size and number seem to increase passing from exponential (Fig. 1F) to stationary (Fig. 1G) cell growth phase.

**Figure 2:**
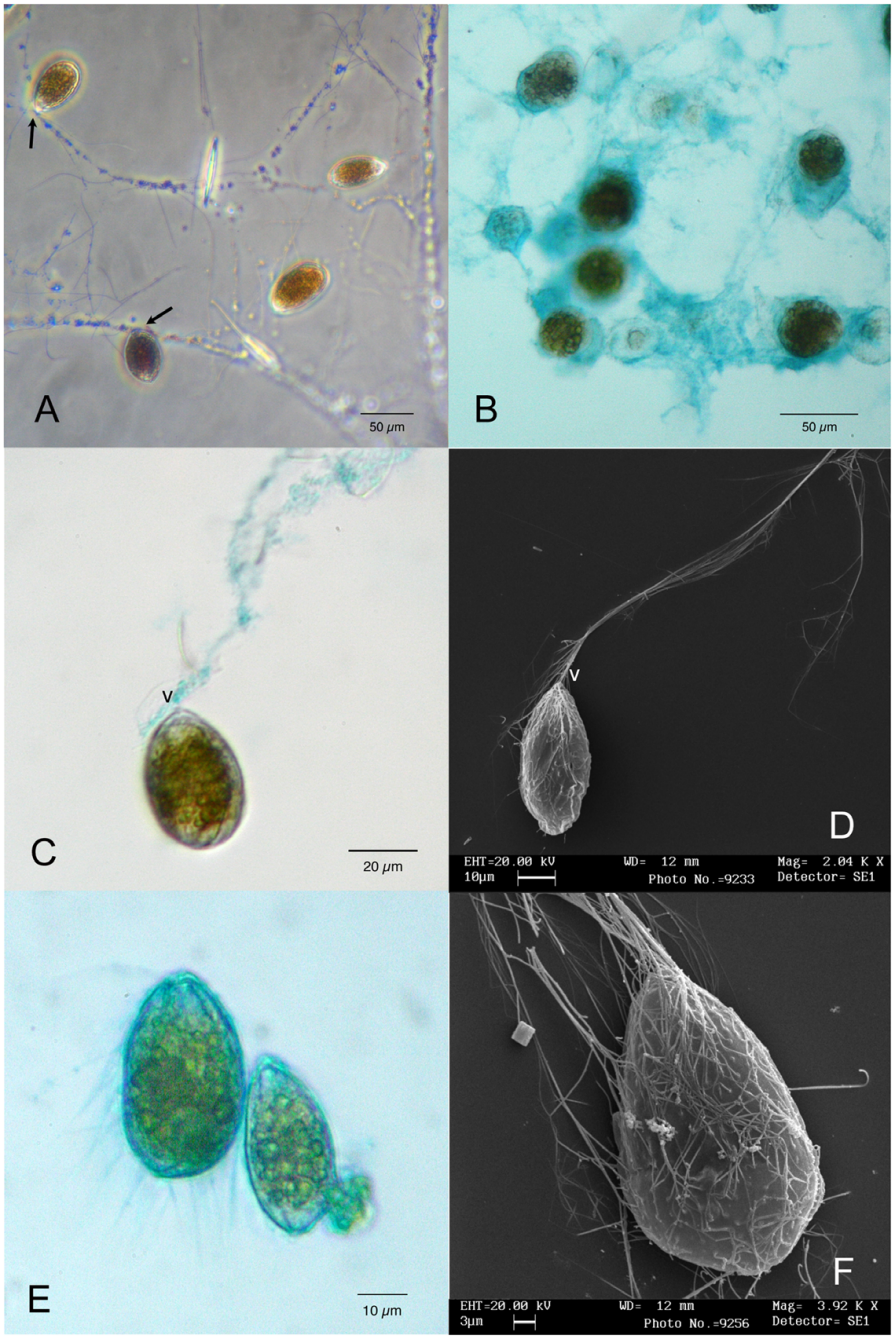
*Ostreopsis* cf. *ovata* mucilage network: Light and scanning electron microscopy. (A) Cells attached to a complex network of tiny filaments. They appear to be anchored to the network always by their ventral side (arrows). Phase contrast microscopy. Scale bar 50 µm. (B) Cells stained with Alcian Blue 8GX. The staining reveals the abundant presence of acidic polysaccharides around cells, embedding them in a continuous matrix. Bright field microscopy. Scale bar 50 µm. (C). Single cell stained with Alcian Blue 8GX. A long filament departs from its ventral side (v) and it is partially stained. Interference contrast microscopy. Scale bar 50 µm. (D) Single cell with a long filament departing from its ventral side (v) observed by scanning electron microscopy. The filament appears to be formed by several thinner filaments. Scale bar 10 µm. (E) Glutaraldehyde fixed cells stained with Alcian Blue 8GX showing positive reaction of cell contour, peripheral cytoplasm and ventral area. Blue stained thin filaments depart from cell surface. Scale bar 10 µm. (F) Cell observed by scanning electron microscopy showing numerous trichocysts extruded through thecal pores over the whole cell surface. Scale bar 3 µm.

Both in nature and in culture cells are embedded in a transparent matrix forming a continuous mucilaginous sheet (Figs. 2A–2F), which covers benthic surfaces (macroalgae, pebbles and rocks) and also the bottom of culture flasks. Often amorphous aggregates detach from the bottom, floating in the water column. Phase contrast microscopy shows that cells are connected to a complex and intricate network of tiny filaments (Fig. 2A). Cells are anchored to filaments from their ventral side, and are able of small rotatory movements around the point of attachment. In the field, also bacteria and diatoms are found to adhere to the filamentous network (Fig. 2A). Alcian Blue specific staining for sulphated and carboxylated polysaccharides [45] reveals the presence of abundant amorphous polysaccharidic material around cells (Fig. 2B): this stuff forms a transparent sheath embedding cells in larger aggregates connected by mucilaginous strands to other aggregates. The filaments, which arise from the cell ventral end and form the filamentous network (see Fig. 2A) are partially stained, too, showing polysaccharidic material on their surface (Fig. 2C); in fixed cells also the peripheral cytoplasm and the cell surface are stained, and many thin filaments departing from it become visible (Fig. 2E).

### Scanning Electron Microscopy

**Figure 3:**
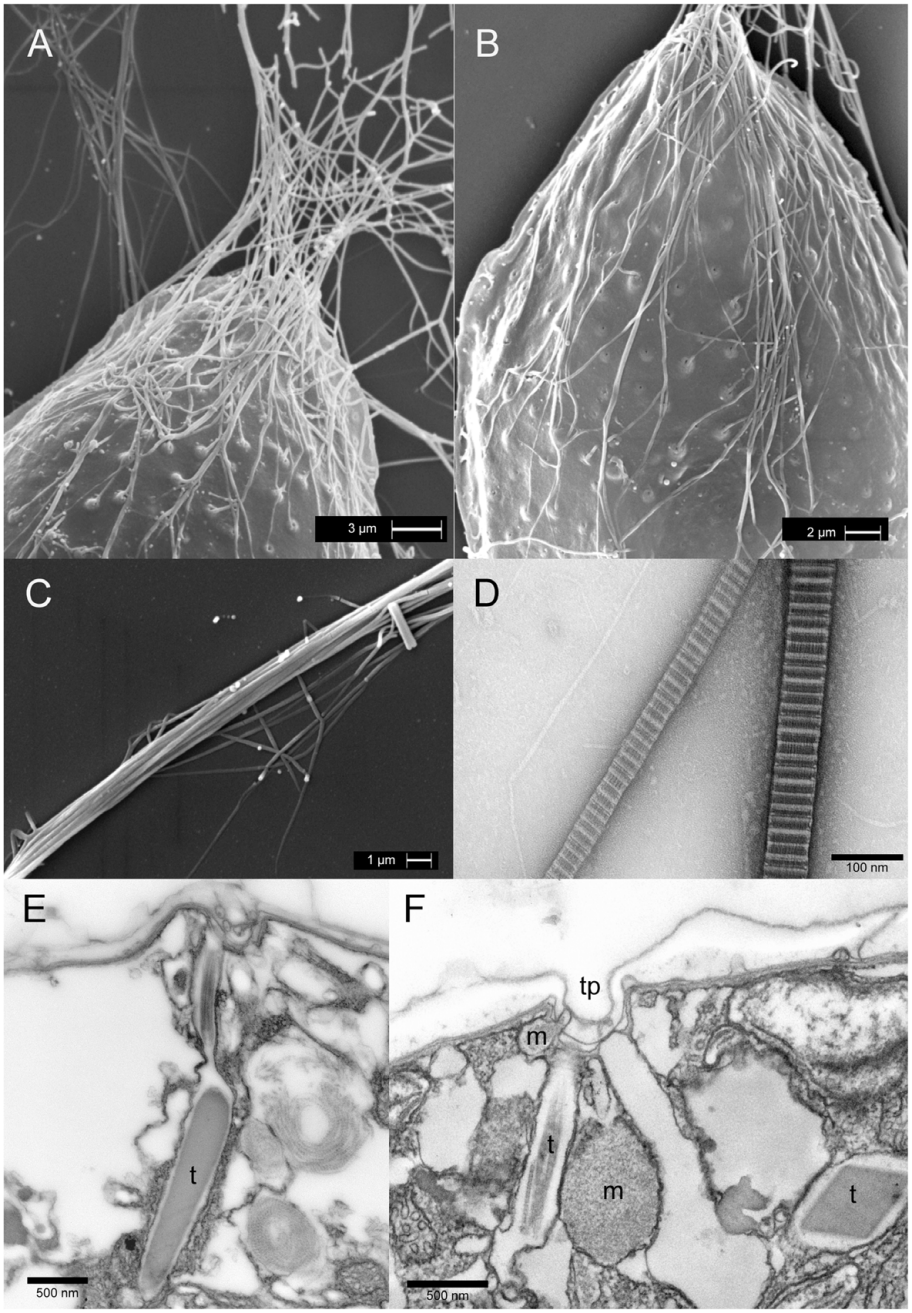
*Ostreopsis* cf. *ovata* trichocysts: Scanning and transmission electron microscopy. (A) Scanning electron microscopy. Cell ventral end: numerous trichocysts extruded through thecal pores converge at the ventral end of the cell where they coalesce together forming a single thicker filament (see also Figs 2D, 2F and 3D). Scale bar 3 µm. (B) Scanning electron microscopy. Trichocysts emerging through thecal pores distributed over the whole cell surface. Most trichocysts are directed towards the ventral end of the cell. Scale bar 2 µm. (C) Scanning electron microscopy. Bundle of trichocysts forming a filament (see also Fig. 2D). Scale bar 1 µm. (D) Transmission electron microscopy: negative staining Trichocyst shafts: they show a banded appearance with major bands period of 68 nm. Scale bar 100 nm. (E) Transmission electron microscopy. Peripheral cytoplasm section showing a trichocyst (t) with crystalline core and terminal fibres perperdicular to the amphiesma. Fixation 1. Scale bar 500 nm. (F) Transmission electron microscopy. Peripheral cytoplasm section showing a thecal pore in connection with a trichocyst (t) (only terminal fibres are visible), two mucocysts (m) with granular content and an the empty sac of an extruded trichocyst. Fixation 1. Scale bar 500 nm.

Thecal plates show a smooth surface and present many sparsely scattered pores (Figs. 3A, 3B). Pores (0.15–0.20 µm wide) appear to be surrounded by elevated areolae (diameter 0.6–0.9 µm). Sutures between thecal plates cannot be clearly distinguished, as hidden by amphiesmal membranes and extracellular mucilage material, previously evidenced by Alcian Blue and preserved by SEM fixation. In all growth stages most cells show a great number of trichocysts extruded through thecal pores (Figs. 3A, 3B). Most of them are oriented in the same direction and converge at the ventral end of the cell, where they become parallel and join together in proximity of the ventral pore (Figs. 2D, 2F, 3A, 3B): here they form a long multi-thread filament about 1 µm thick (Fig. 3C). These filaments (Fig. 2D) deriving from the assemblage of parallely oriented trichocysts (Figs. 2F, 3C) are the constituents of the filamentous network observed by light microscopy (Fig. 2A).

#### Transmission electron microscopy trichocysts and mucocysts

Direct preparations of whole cells by negative staining confirm that filaments extruded through thecal pores are trichocysts shafts: they present the typical banded appearance, with major bands period of 68 nm. Their width ranges between 65 and 80 nm (Fig. 3D).

In sections, numerous resting trichocysts with an electron dense core (length: 1.6–2.0 µm; max width: 270–320 nm) and terminal twisted fibres can be observed; they are perpendicular to the cell surface and open into thecal pores (Fig. 3E). Also mucocysts are found in the peripheral cytoplasm (Fig. 3F). They are surrounded by one membrane and are flask-shaped with an oval rounded body containing finely granular material and a short neck connected to a thecal pore. Arrays of trichocysts and mucocysts, ready to discharge their content outside the cell, are often found beneath the same thecal pore. In Fig. 3F a small and a large mucocyst with granular amorphous material, a trichocyst (only terminal fibres are visible in the section) and an empty membrane envelope of an already discharged trichocyst can be observed beneath a thecal pore. Thus, cells seem to be able to discharge through the same pore long trichocyst shafts and amorphous mucocyst material.

#### Fibrous vesicles and pusule

**Figure 4:**
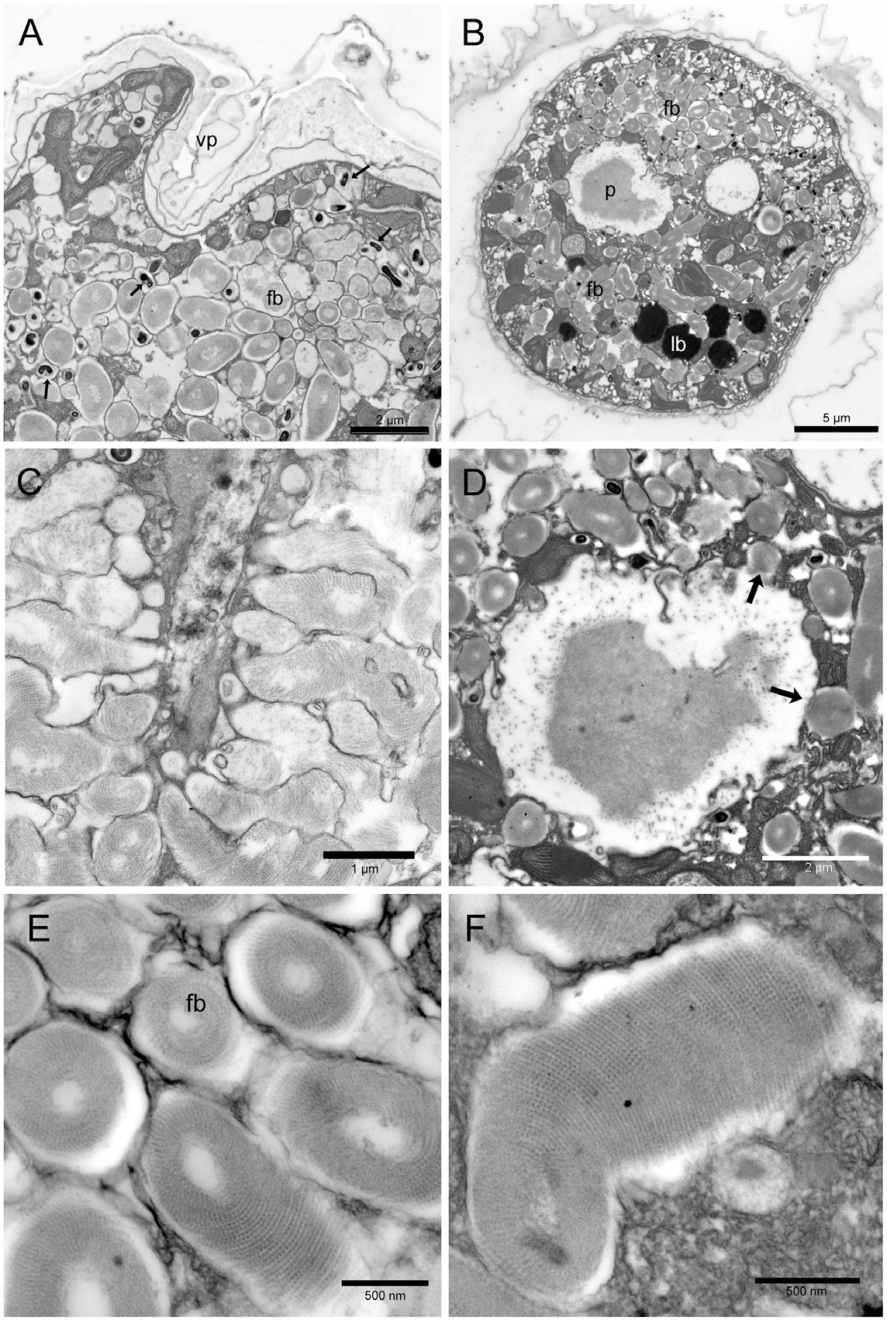
*Ostreopsis* cf. *ovata* fibrous bodies and pusule: Transmission electron microscopy. (A) Oblique section of the ventral end of a cell showing the ventral pore (vp) and numerous fibrous bodies (fb) which occupy most cytoplasm. Smaller more electron dense fibrous bodies are also visible (arrows). Fixation 1. Scale bar 2 µm. (B) Transverse section of the cell showing the pusule chamber (p), containing electron dense material, surrounded by numerous fibrous bodies (fb). Lipid bodies (lb) are also visible. Fixation 1. Scale bar 5 µm. (C) Section of an elongated structure (possibly the pusule canal) with numerous fibrous bodies perpendicular to it: some of them seem to discharge their content into the canal. Fixation 2. Scale bar 1 µm. (D) Transverse section of the pusule chamber containing electron dense fibrous and amorphous material. Some fibrous bodies seem to discharge their content into the chamber (arrows). Fixation 1. Scale bar 2 µm. (E) Fibrous bodies (fb) in transverse and oblique section: they show an organized structure made of spirally arranged filaments packed around an electron transparent core. Fixation 1. Scale bar 500 nm. (F) Fibrous body in longitudinal section. Fixation 1. Scale bar 500 nm.

A large number of fibrous material containing vesicles is found in the ventral part of the cell (Figs. 4A–4F). They are delimited by two membranes. Fibrous material is made of spirally arranged tiny filaments packed around an electron transparent core to form slightly curved fusiform structures (1.5–3.0 µm long and 0.5–0.7 µm wide). The filaments are made of regularly spaced smaller subunits (12–14 nm wide, with a periodicity of 25 nm) (Figs. 4E–4F). Fibrous vesicles are radially arranged around a large rounded chamber delimited by two membranes and containing amorphous and fibrillar material (Fig. 4B, 4D). In some sections fibrous vesicles seem to open into the large rounded chamber (Fig. 4D arrows) and into a narrower canal (Fig. 4C). The whole structure is likely to represent the pusule. The large chamber can be observed also by light microscopy, where it appears to be connected by a narrow canal to the ventral end of the cell (Fig. 1D). The presence of amorphous and fibrillar material inside the rounded chamber suggests that fibrous vesicles material could be discharged into the large collecting chamber and then released outside the cell through the canal and the ventral pore. This is supported by Alcian Blue staining showing the release of amorphous polysaccharidic material in proximity of the ventral pore (Fig. 2C). Numerous small more electron dense structures are often associated with fibrous vesicles (Fig. 4A arrows). In transverse section, they are rounded (diameter 200–210 nm), while longitudinally, they show an elongated curved shape. Since these structures are always associated with fibrous vesicles and present the same arrangement around the pusule large chamber, they could represent the same fibrous material in a more condensed form.

#### Lipid bodies and accumulation bodies

**Figure 5:**
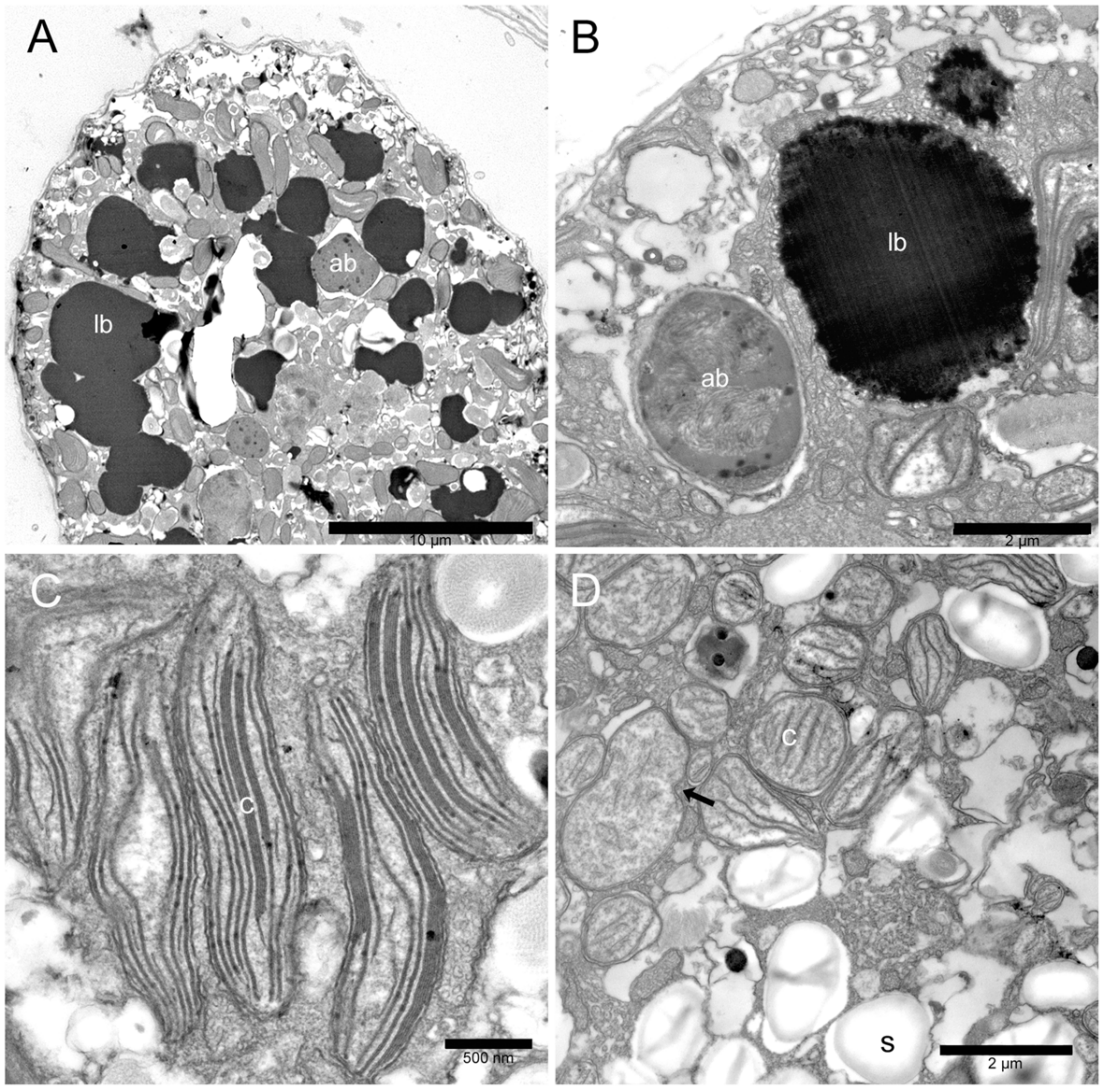
*Ostreopsis* cf. *ovata* lipid droplets, accumulation bodies, and chloroplasts: Transmission electron microscopy. (A) Cell section with numerous large electron dense lipid droplets (lb) in the peripheral cytoplasm. An accumulation body (ab) is visible. Fixation 2. Scale bar 10 µm. (B) Lipid droplet (lb) and accumulation body (ab) in the peripheral cytoplasm. Lipid droplets are highly osmiophilic and present an irregular contour with small drops coalescing together. The accumulation body contains membranous and fibrillar material and appears to be surrounded by smooth endoplasmic reticulum. Fixation 1. Scale bar 2 µm. (C) Elongated chloroplasts (c) in the peripheral cytoplasm. Fixation 1. Scale bar 500 nm. (D) Small rounded plastids (c) with single thylakoids and girdle lamella in the inner part of the cell. A dividing plastid is visible (arrow). Starch granules (s). Fixation 1. Scale bar 2 µm.

Cell cytoplasm presents also two different kinds of bodies: highly osmiophilic electron dense bodies and accumulation bodies similar to those observed in other dinoflagellates [46]. Osmiophilic electron dense bodies (Figs. 5A, 5B) are numerous and present an irregular rounded shape; their size is highly variable ranging from 1.7 to 12 µm, and they appear generally larger in stationary and senescent growth phases. They are not surrounded by membranes and their margin is rather irregular. Their outer region is less electron dense and appears to be formed by small coalescing droplets, while the inner part is compact and completely dark (Fig. 5B). Their highly osmiophilic nature and the strong fluorescence exhibited after Nile Red staining under epifluorescence microscopy (Figs. 1F,1G) suggest that they are mainly composed by lipids. They may represent a significant part of cell volume, as seen by TEM sections (Fig. 5A), and light (Fig. 1E) and epifluorescence microscopy (Figs. 1F, 1G).

Accumulation bodies are generally present in the central part of the cell; they are rounded (generally with a diameter of 2.0–3.5 µm, but reaching also 6 µm in some cases) and show electron dense fibrillar and membranous material inside; they are surrounded by smooth endoplasmic reticulum (Fig. 5B). Their position and size suggest that they correspond to the small yellow fluorescing bodies observed by epifluorescence microscopy (Fig. 1B).

#### Plastids and other organelles

Two different types of plastids are found in all stages of cell growth (Figs. 5C, 5D). Plastids in the inner part of the cell are generally smaller with a rounded or ellipsoidal shape. Some do not present thylakoids but only a girdle lamella-like structure. Others show, in addition, few single sparse thylakoids, which present a parallel arrangement when their number increases (Fig. 5D). Dividing plastids with a central constriction are frequently observed in the central part of the cell (Fig. 5D, arrow). Peripheral chloroplasts are elongated and considerably larger (up to 2.5 µm long): they are radially arranged and show thylakoids in stacks of 3 (sometimes also 5) (Fig. 5C). No girdle lamella is found. All plastids appear to be surrounded by an envelope formed by two very closely packed membranes. Starch granules are present in the cytoplasm (Fig. 5D).

Mitochondria with tubular cristae are numerous and smooth endoplasmic reticulum, continuous with nuclear envelope outer membrane, appears particularly developed (Fig. 5D).

### HR LC-MS Analyses

**Figure 6:**
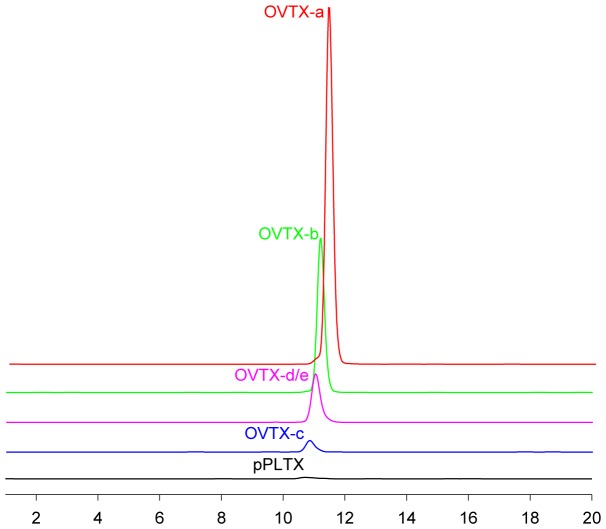
Extracted ion chromatograms (XIC) of the principal components of the O. cf. ovata toxin profile. XIC were obtained by selecting the [M+2H-H2O]^2+^ and [M+H+Ca]^3+^ ion clusters of palytoxin and ovatoxins contained in their HR full MS spectra.

The Adriatic *O.* cf. *ovata* strain was cultured under the conditions as previously reported. Culture aliquots were collected on day 10, 18 and 25 after the establishing of the culture, namely at the end of exponential, stationary and senescent growth phases, respectively. Cell pellets were separately extracted and crude extracts were directly analysed by HR LC-MS to evaluate their toxin profile. Fig. 6 shows the extracted ion chromatograms (XIC) of individual components of the toxin profile. Most toxins overlapped under the used LC conditions but unambiguous identification of toxins and accurate quantitation was possible through the use of high resolution MS and additional confirmation provided by MS^2^ experiments [44]. Toxin content of *Ostreopsis* cultures in different growth phases is reported in Table 1. Similarly to most *O.* cf. *ovata* strains analysed so far, these cultures showed to produce mainly OVTX-a and smaller amounts of its analogues (OVTX-b, OVTX-c and OVTX-d/e). Putative palytoxin was present only in very minute amounts, lower than 0.1 pg/cell. OVTX-f was not detected. The toxin content increased with aging of culture; in particular, cells in senescent phase (day 25) had about twice the toxin content of those in stationary phase (day 18).

**Table 1:** Individual and total toxin content (pg/cell) of Adriatic *O.* cf. *ovata* cultures collected at different growth phases.

Sample #	pPLTX	OVTX-a	OVTX-b	OVTX-c	OVTX-d+e	OVTX-f	Total
Exponential phase	0.03	7.5	3.6	0.6	1.6	nd	13
Stationaryphase	0.04	9.5	4.6	0.9	2.3	nd	17
Senescentphase	0.08	20	9.3	1.5	4.4	nd	35

#### Raman microspectroscopy raman spectrum of palytoxin

**Figure 7:**
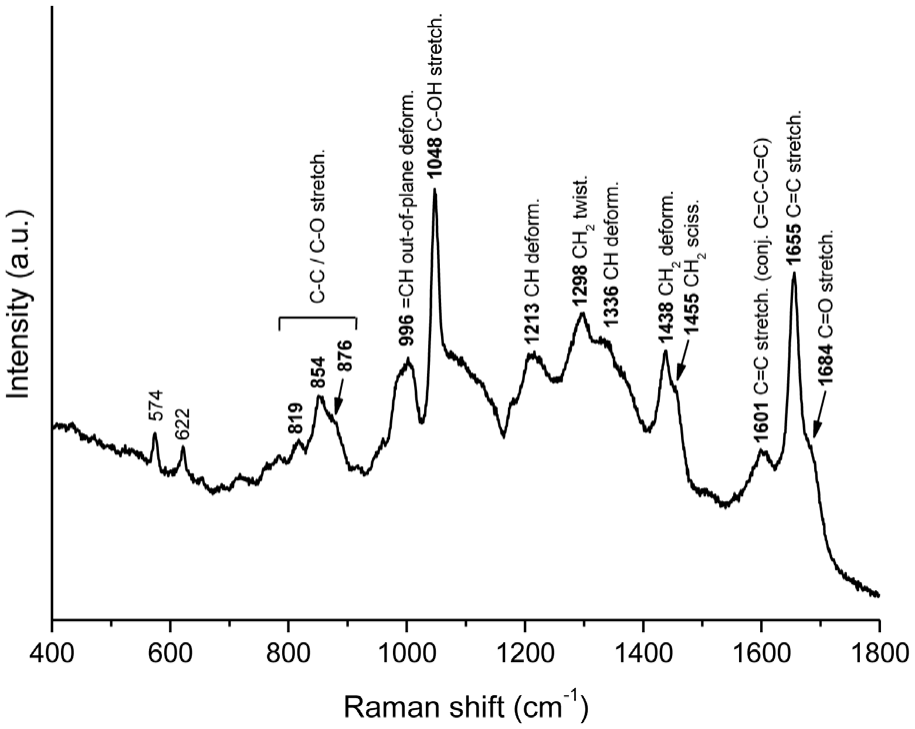
Raman spectrum of palytoxin. 400–1800 cm-1 region of the Raman spectrum of palytoxin (solid); bands are labeled with the corresponding Raman shifts and with tentative vibrational modes assignments. Excitation wavelength is 785 nm.

The obtained Raman spectrum of palytoxin is shown in Fig. 7. All the bands were tentatively assigned, according to literature [47], [48], to a group vibration. The assignments are in agreement with the proposed palytoxin structure [49], [50]. The intense band at 1655 cm^−1^ and the band at 1601 cm^−1^ can be attributed to the stretching vibration of isolated C = C and conjugated C = C-C = C groups, respectively. Another characteristic feature is the intense band at 1048 cm^−1^, tentatively attributed to the stretching vibration of C-OH groups, which constitute a large part of palytoxin structure. All the bands in the 1200–1500 cm^−1^ region are assigned to C-H deformation vibrations, and in particular the bands at 1455 and 1298 cm^−1^ can be attributed to CH_2_ twisting and CH_2_ scissoring modes from long alkyl chains, respectively [47], [48].

#### Raman spectra of living O. cf. ovata and carotenoids distribution within the cell

**Figure 8:**
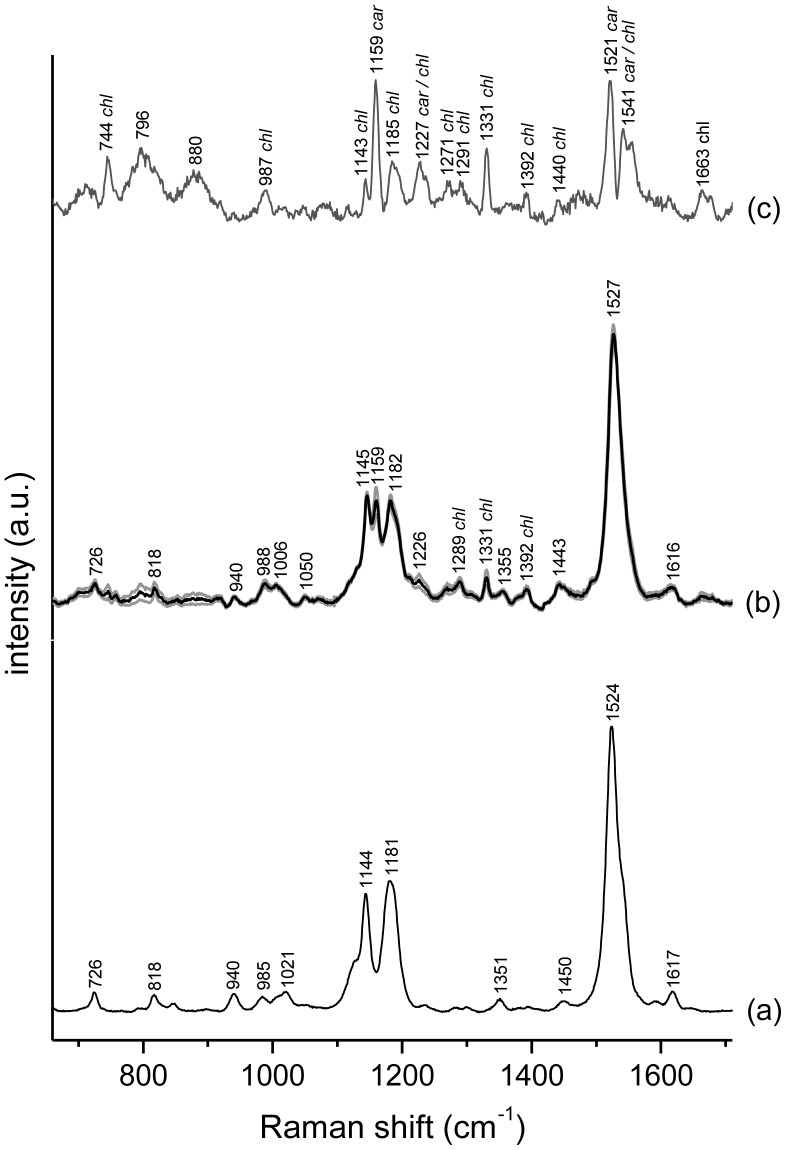
Raman spectra of peridinin and living *O.* cf. *ovata* cells. 400–1800 cm-1 region of (a) the Raman spectrum of peridinin (solid); (b) average normalized Raman spectrum (in black) plus and minus the intensity S.D. (in grey) of a set of 24 spectra collected from 24 different *O.* cf. *ovata* cells; (c) S.D. of the Raman intensity for the same set of 24 cells, plotted with a magnified intensity scale. In all spectra, bands are labelled with their Raman shift. In (b) and (c) bands assigned to chlorophyll are labelled as chl, whereas in (c) bands assigned to carotenoids are labelled as car. Excitation wavelength 785 nm.

The average Raman spectrum of a set of 24 cells is shown in Fig. 8b, together with the intensity standard deviation (grey lines in Fig. 8b and Fig. 8c). The spectrum is dominated by spectral features typical of carotenoids [51], [52], with a minor contribution from chlorophylls [42], [53], [54]. In particular, the spectrum in Fig. 8b is dominated by the characteristic features of peridinin [55], reported in Fig. 8a as reference. It presents a relatively small standard deviation (S.D.), indicating a spectral homogeneity in the set of cells investigated.

Besides the weak chlorophyll bands (which are best observed in the S.D. spectrum in Fig. 8c), small but significant differences between spectra of peridinin (Fig. 8a) and *O.* cf. *ovata* (Fig. 8b), such as a sharp band at 1159 cm^−1^ and a slight shift of the most intense band, indicate that carotenoids other than peridinin are also present in the microalgal cells.

**Figure 9:**
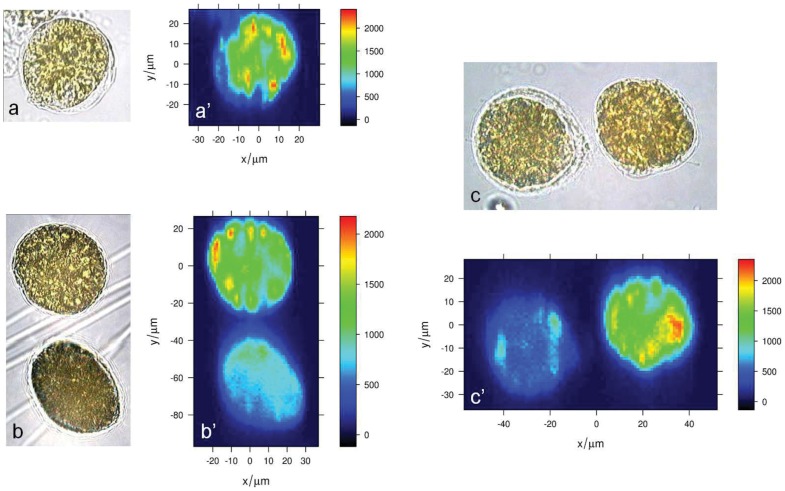
Bright field transmission micrographs and Raman maps of fixed cells at different growth phases. (a–c) bright field transmission micrograph of fixed *O.*cf. *ovata* cells in the (a) exponential, (b) stationary and (c) senescence phase, and (a’–c’) the corresponding Raman map depicting carotenoids concentration based on the un-normalized intensity of the ν_1_ carotenoid band at 1527 cm^−1^.

Because of their intense signal, Raman microspectroscopy allows carotenoids to be mapped within microalgal cells [43], [56], [57], [58]. Carotenoids distribution in *O.* cf. *ovata* cells is mapped by imaging the intensity of the intense carotenoid band at 1527 cm^−1^ (Fig. 9) in baseline-corrected unnormalized Raman maps. According to the Raman image in Fig. 9, carotenoids appears to be relatively diffuse in the whole cell, with spots located toward the border of the cell having a higher local carotenoids concentration. Moreover, such pattern in the intracellular carotenoids distribution appears to remain similar through all the cell growth phases.

Despite *O.* cf. *ovata* is known to produce palytoxin and several other structurally related toxins, no evident bands, which could be attributed to these toxins, were observed in its Raman spectra.

#### Raman spectroscopy and imaging of depigmented cells

**Figure 10:**
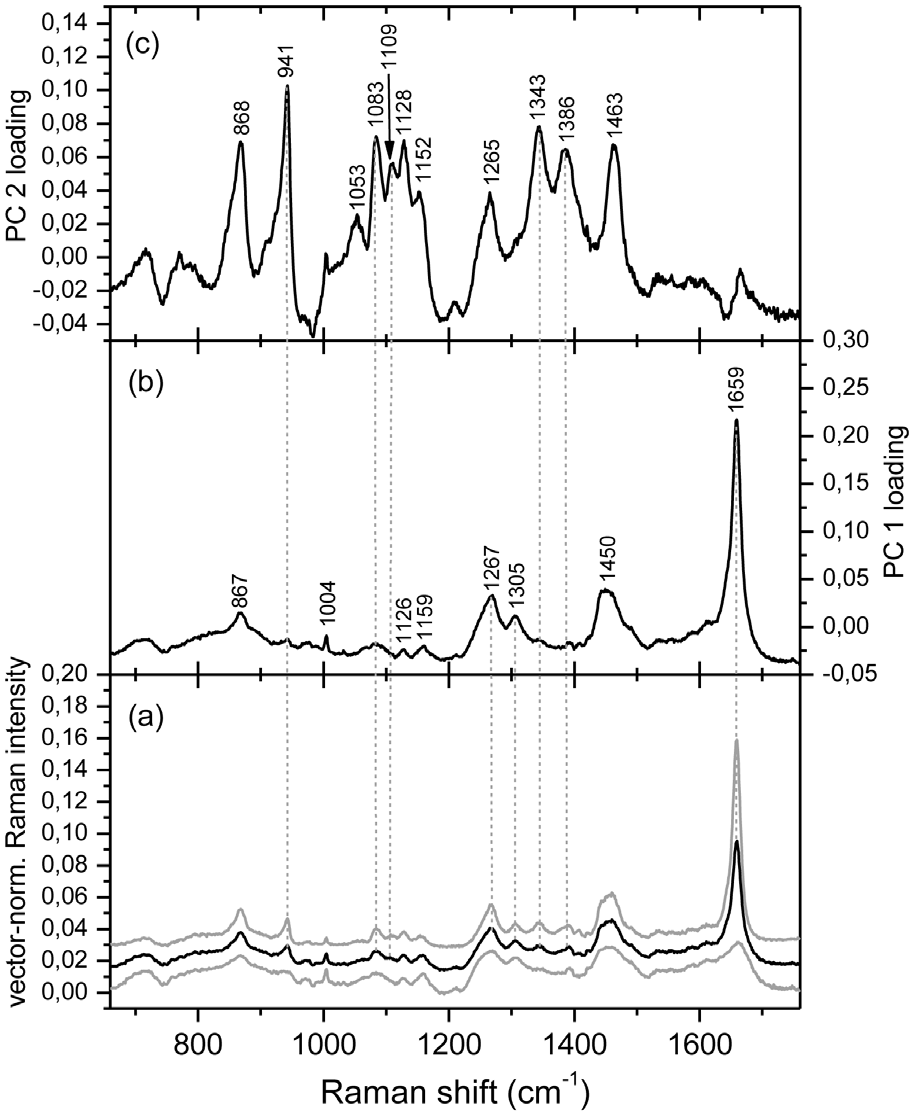
Raman spectra of a map of a fixed and depigmented O. *ovata* cell in the exponential phase. (a) average (in black) and st.dev. (in grey) of the vector-normalized Raman spectra of a map of a fixed and depigmented *O.* cf. *ovata* cell in the exponential phase consisting of 1681 (41×41) spectra (see Fig. 11); (b) loadings for the first principal component PC 1 and (c) loadings for the second principal component PC 2. Excitation wavelength 785 nm.

To better investigate chemical constituents other than carotenoids and chlorophylls, which are present in *O.* cf. *ovata* cells, pigments were removed upon washing with an acetone:hexane solution, after fixation. Raman maps were then collected from depigmented cells. Fig. 10a shows the average spectrum of a map collected from a depigmented cell in the exponential phase, where the carotenoids and chlorophyll bands have disappeared. Without the overwhelming signal due to carotenoids, other bands can be observed, which are due to unsaturated lipids and to amylose and amylopectin, starch components. The presence of such compounds is evident in the loadings of the first and second principal components PC 1 and PC 2 (Fig. 10b, 10c) as calculated from a principal component analysis (PCA). The loadings of the first component PC 1 (Fig. 10b) have typical features of Raman spectra of poly-unsaturated lipids, and in particular of poly-unsaturated fatty acids (PUFA) or their esters [59], [60], [61], whereas the second component PC 2 (Fig. 10c) shows the characteristic bands of starch [60], [62]. The high relative intensity of the unsaturated lipids bands, with respect to the phenylalanine ring mode at 1004 cm^−1^ which is characteristic of proteins, indicates that such lipids are present in relatively high concentration in *O.* cf. *ovata* cells. The loadings of the first principal component PC 1 (Fig. 10b) have frequencies and relative intensities, which are well matched to those of docosahexaenoic acid and one of its esters [59], [63], [64].

**Figure 11:**
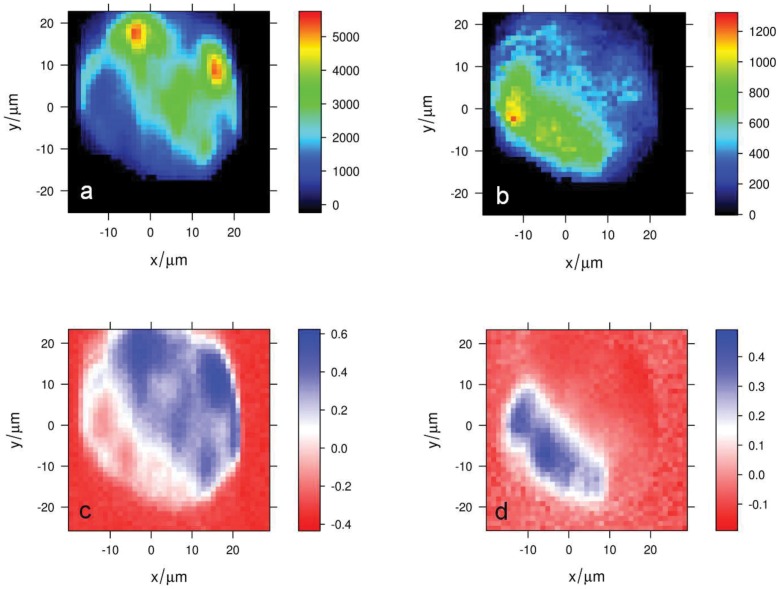
Raman maps of a fixed and depigmented *O.* cf. *ovata* cell in the exponential phase. (a) poly-unsaturated lipids concentration based on the un-normalized intensity at 1559 cm-1 (C = C stretching); (b) starch concentration based on the un-normalized intensity at 941 cm-1 (α-1,4 glycosidic bond C-O, C-O-C and C-O-H vibrations), together with the score maps of (c) the first principal component PC 1 and (d) the second principal component PC 2. The average Raman spectrum of this map, together with the loadings of the PC 1 and PC 2 are shown in figure 10.

Raman images depicting the distribution of starch and poly-unsaturated lipids, based on the intensity of characteristic bands for these two substances, are shown in Fig. 11a and 11b), together with the score maps for the first and second principal components PC 1 and PC 2 (Fig. 11c, 11d). Both score and intensity maps show that poly-unsaturated lipids are concentrated in spots located at the cell border, whereas starch is more homogeneously distributed throughout the cell. Similar distributions of poly-unsaturated lipids and starch are observed in depigmented cells in the stationary and senescence phases (see Supplementary Information; Fig. S2).

## Discussion

The species is identified as *O.* cf. *ovata*. To date, resolving the taxonomy of *Ostreopsis* species based only on morphology has been difficult due to the morphological variability of both field material and cultured specimens [9]. Furthermore, none of the original isolates from tropical areas from which *Ostreopsis* species were described has yet been sequenced for the genotype assignment. Given that for many dinoflagellate genera there is a discrepancy between morphology-based taxonomy and genetic studies [9], the species names applied to strains of different genetic lineages must be treated with caution. It is therefore advisable to use the designation *O.* cf. *ovata* when referring to isolates from the Mediterranean Sea [3].

The ultrastructural and cytochemical analysis of *O.* cf. *ovata* extracellular mucilage reveals unique features never described before in microalgae. *Ostreopsis* mucilage shows a complex structure, formed by a network of long fibres, deriving from trichocysts extruded through thecal pores, and by an amorphous matrix of acidic polysaccharides, stained by Alcian Blue. Mucilages consisting of hygroscopic lattice-like polymers of carbohydrate are a common feature of many microalgae, where they seem to exert various functions, as defence against grazing, increased buoyancy and metabolic self-regulation [65], [66]. Exuded polysaccharides forming a matrix of transparent exopolymer particles (TEP) can be visualized by Alcian Blue [37], [67]. Mucilaginous aggregates are common in dinoflagellates: large thecate species embedded in thick viscous mucus material are often found in the water column [68] and some non-toxic species, as *Gonyaulax hyalina* [69], *G. fragilis* [70] and *Lepidodinium chlorophorum* [71], are known to give rise to gelatinous planktonic blooms, with high amounts of mucilage, deeply stained by Alcian Blue [72], [73]. *Ostreopsis ovata, Gambierdiscus toxicus, Coolia monotis* and other benthic dinoflagellates are known to produce copious amounts of mucilage, which has a role in the attachment of these species to surfaces with the formation of transparent films embedding many cells [8], [16], [20]. *Ostreopsis* films appear particularly resistant [74]. Our observations show that *Ostreopsis* mucilage is formed, as expected, by acidic polysaccharides, and by a very high number of trichocysts sticking together to form a complex network of filaments. *O.* cf. *ovata* trichocysts shafts show the typical structure and size (period 68 nm, width 65–80 nm) of dinoflagellate trichocysts, which present a period from 65 to 85 nm and a width from 40 to 180 nm [75], [76].

A role of trichocysts as structural elements in microalgal extracellular mucilage is completely new and has never been observed before. Trichocysts are ejectile organelles found in many dinoflagellates, microalgae and protists. Although their function has not been completely clarified yet, it has been generally related to defence against grazing, excretion or prey capture. The presence of a network of many trichocyst filaments in the mucilage embedding *O.* cf. *ovata* cells could contribute to provide more mechanical resistance to the mucilage, copiously produced by this species: this could represent an advantage for *Ostreopsis* in the colonization of different surfaces and could explain, at least in part, its rapid proliferation without being dispersed by hydrodynamism. Furthermore, a more complex resistant mucilage structure could be a better defence against grazing, and the trichocysts web could give a way to trap and surround larger predators, as observed by Barone [24]. Recently it has been put in evidence a role of toxic mucus traps in prey uptake by *Alexandrium pseudogonyaulax* [77]: a similar mechanism could be hypothesized also for *Ostreopsis* in view of its copious mucus production and the release of toxins in the medium observed in culture experiments [17]. In addition mucilage plays an important role in the dynamics of *Ostreopsis* blooms characterized by a benthic initial phase with the formation of brownish films (mats) on surfaces followed by their detachment from the bottom and resuspension in the water column by the mechanical action of waves [78]. We observed abundant *Ostreopsis* mucilaginous floating aggregates during blooms in the Gulf of Trieste in 2009 and 2011 (unpublished data). Mucilage contributes to the buoyancy of planktonic aggregates and may favour the formation of toxic aerosols.

TEM observations show that the polysaccharidic component of *Ostreopsis* mucilage derives from two different sources: one related to the release of pusule fibrous material at the ventral end of cell and the other to mucocysts ejected through thecal pores. Besada et al. [20] recognized for the first time the abundant presence of vesicles containing spirally coiled fibres as a characteristic feature of *Ostreopsis*, *Coolia* and *Gambierdiscus*, and suggested that these vesicles, aggregated around a canal, represented the pusule and discharged their content outside the cell through the ventral pore. Our observations support this hypothesis: Alcian Blue staining clearly shows filaments of polysaccharidic material discharged in proximity of the ventral pore (Fig. 2C). In addition, SEM images (Fig. 2D) show that trichocysts stick together to form a thicker filament (Figs. 2D, 3C) near the ventral pore (Fig. 2F) and this could be related to the secretion of polysaccharidic material from the ventral pore. Further mucilage material is likely to derive also from mucocysts placed with trichocysts beneath thecal pores. Mucocysts are extrusive organelles related to mucilage secretion found in many dinoflagellates [79]. *O.* cf. *ovata* mucocysts are flask shaped with granular content, similar to those observed in *Prorocentrum lima* and *P. maculosum,* which are lacking of trichocysts [80]. Also these two benthic dinoflagellates possess, like *Ostreopsis*, an elaborate pusule system and the pusule canal is often filled with fibrillar material, probably representing mucilage material to be excreted outside the cell.

Other interesting features of *O.* cf. *ovata* are given by chloroplasts, accumulation bodies and cytosolic lipid droplets. Plastids resemble those observed in *Gambierdiscus toxicus*, where two kinds of chloroplasts are found: peripheral elongated tibia-shaped chloroplasts and smaller inner plastids with different thylakoid arrangements [21]. It is likely that they represent different stages of plastid differentiation: in the centre of *Ostreopsis* cells, proplastid-like structures (some of them also dividing) are found near small chloroplasts with few thylakoids, while large fully differentiated chloroplasts are present mainly in the peripheral cytoplasm. In unicellular algae with one or few chloroplasts, chloroplast division occurs once per cell cycle prior cytokinesis to maintain the number of chloroplast per cell [81]. Chloroplast division has been observed by TEM also in dinoflagellates [82]. Proplastids in microalgae are generally found in facultative heterotrophic unicellular algae as result of chloroplast dedifferentiation when cells are heterotrophycally grown in the dark [83]. In *O.* cf. *ovata* and *G. toxicus* proplastid-like structures and differentiating plastids are found in the inner cytoplasm of photosynthetically growing cells. In this case it could be hypothesized that in these benthic dinoflagellate species the chloroplast number per cell is maintained at least in part by division of proplastid-like structures and their subsequent differentiation, as it occurs in some macroalgae and in meristematic tissues of higher plants [81]. In *O.* cf. *ovata* the chloroplast envelope presents two membranes rather than three, as generally observed in peridinin containing dinoflagellates [84], although two membrane envelopes are only rarely found in dinoflagellates chloroplasts [85]. Also in *Gambierdiscus toxicus*, chloroplasts can present a two-membrane envelope [21]. Pigment analysis by Raman microspectroscopy revealed the presence of peridinin as predominant carotenoid in living *O.* cf. *ovata* cells, confirming previous observations in *G. toxicus* [21], *Coolia canariensis, C. monotis* and *O. ovata* [86], which revealed similar pigment profiles.

Accumulation bodies seen in TEM sections and by epifluorescence microscopy under blue excitation show a great resemblance with similar structures observed in other dinoflagellates, as *Prorocentrum lima* and *P. maculosum* [46], *Lingulodinium polyedrum* [87], [88], *Amphidinium poecilochroum* [89] and other species. Dinoflagellate accumulation bodies, containing electron dense material, fibrous material and membranous material, and often surrounded by smooth endoplasmic reticulum, are considered the dinoflagellate equivalent of lysosomes, in view of their acid phosphatase activity and positive reaction with the periodic acid/Schiff reagent [46].

Cytosolic lipid droplets, particularly abundant in *O.* cf. *ovata* are a common feature of most eukaryotic cells [90], and some algae can accumulate them in a large number, as storage reserve in response to nutrient limitation or stress [91], [92]. Lipid bodies are commonly found also in dinoflagellate cells, which can accumulate lipids (more than 20% of dry weight in *Crypthecodinium cohnii* [93]). Nile Red is a specific fluorescent stain for lipids, used to localize and quantify neutral lipid droplets within cells [39]. Neutral lipids give yellow fluorescence and polar lipids red fluorescence. In *O.* cf. *ovata,* neutral lipid droplets are particularly abundant in peripheral cytoplasm, in all growth phases, as clearly evidenced by epifluorescence and TEM. The abundant presence of lipid droplets has been also shown by Nile Red in other dinoflagellates, as *Crypthecodinium cohnii* [94], *Alexandrium andersoni*, *A. minutum* and *Karlodinium veneficum* [95]. Such lipids are however washed out by acetone:hexane treatment used to get rid of carotenoids prior to Raman measurements (see Supplementary Information; Fig. S1). In spite of acetone:hexane treatment Raman data show the presence of poly-unsaturated lipids, suggesting PUFA esters, and in particular a docosahexaenoic acid ester as the prevalent compound [61]. Such a compound is frequently found in dinoflagellates [96], as part of neutral as well as polar lipids. The present data, however, do not allow the precise identification of the DHA-containing lipid, which could be a phospholipid, a glycolipid or even a free fatty acid.

*O.* cf. *ovata* is known to produce toxins belonging to the palytoxin family [12]. Total toxin content of the examined Adriatic culture increased with aging of culture: cell pellets collected in senescent phase (day 25) contained about twice the toxins detected at the stationary phase (day 18); so these toxins accumulate when cells are not in duplication anymore. This behaviour is in agreement with the observation of Pistocchi et al. [97] who reported that *O.* cf. *ovata* increases toxin production during the progression of growth from exponential to stationary phase. *O.* cf. *ovata* behaves differently than other harmful algal species, such as *Pyrodinium bahamense* and *Alexandrium* spp., which present the highest toxin level during the exponential phase [98], [99].

OVTX-a was by far the major component of toxin profile, representing the 56–58% of the total toxin content, followed by OVTX-b (26–28%), OVTX-d and-e (12–13%), OVTX-c (4–5%) and pPLTX (0.2%). No OVTX-f has been detected. Relative abundance of individual compounds is similar among samples collected at different growth phases. Toxin profile closely resembles that previously reported for most *O.* cf. *ovata* strains [17], [28], [29]. Anyway it has to be noted that some difference in toxin profiles can occur in strains collected at different sites or even at the same site but in different periods; by way of example, natural samples of *O.* cf. *ovata* collected in 2009, in the same area where cells analysed in this study were isolated (Canovella de’ Zoppoli, Gulf of Trieste, Italy), did not contain any pPLTX but only ovatoxins [30]. Even more significant differences have been recently reported in toxin composition of two Adriatic isolates: a strain from Numana that does not produce neither OVTX-b nor OVTX-c [13] and a strain from Portonovo that is dominated by the new OVTX-f [14]. This confirms the variability of toxin profiles and contents also in *Ostreopsis* species. Despite our efforts, such palytoxin-like compounds could not be detected in *O.* cf. *ovata* cells with Raman microspectroscopy, probably due to their low concentration and to their diffuse distribution in the cytoplasm [30].

### Conclusions

The results show new features of *O.* cf. *ovata*. The role of trichocysts, as fibrillar components of extracellular mucilage, is unique and allows the formation of a more structured mucilaginous sheath embedding cells and avoiding their dispersion by hydrodynamic factors. Further studies will be necessary to clarify if mucilage may contribute to enhance toxic effects maintaining highly concentrated spots of cells and toxins, and thus explaining the massive death of marine benthic fauna during *Ostreopsis* blooms [100], [101], [102], [103], [104].

Results provided by Raman spectroscopy and HR LC-MS depicted a more complete picture of secondary metabolites produced by *O.* cf. *ovata*. HR LC-MS analysis showed that the analysed strain had a toxin profile similar to most field and cultured *O.* cf. *ovata* reported so far [17], [28], [29]: it contains OVTX-a as dominant toxin, OVTX-b as second major component, followed by OVTX-d/e, -c and pPLTX. Relative abundance of individual components of toxin profile keeps constant during growth phases and the highest toxin content is reached during the senescent phase, indicating that toxins accumulate even when cells do not duplicate anymore.

Another interesting feature is given by the presence of high metabolic reserves, made mainly by lipids, observed both by TEM and Raman microspectroscopy, as well as by starch, found in all phases of cell growth. The combination of different techniques allows us to give a clearer picture of the metabolites of the harmful *O.* cf. *ovata* Mediterranean/Atlantic clade.

## Supporting Information

Figure S1 ***Ostreopsis*** **cf.** ***ovata*** **depigmented cells stained with Nile Red.** Cells were fixed with 2% paraformaldehyde (5 min at RT), depigmented upon washing 3 times with an 1∶1 acetone:hexane solution for 5 min and stained with Nile Red. Bright field microscopy (A,C); epifluorescence microscopy (B,D). Cells show a weak red and yellow orange fluorescence. Strongly yellow fluorescing lipid droplets are no more visible.
(TIF)

Figure S2 **Raman maps depicting poly-unsaturated lipids and starch concentration of fixed and depigmented** ***O.*** **cf.** ***ovata*** **cells in the stationary and senescence phases.** Raman maps depicting poly-unsaturated lipid concentration based on the un-normalized intensity at 1559 cm^−1^ of a fixed and depigmented *O.* cf. *ovata* cell in the stationary (a) and in the senescence (c) phases; Raman maps depicting starch concentration based on the un-normalized intensity at 941 cm^−1^ of a cell in the stationary(b) and in the senescence (d) phases.
(TIF)
