# Supplementary figures and images for: New Insights on Cytological and Metabolic Features of Ostreopsis cf. ovata Fukuyo (Dinophyceae): A Multidisciplinary Approach
Source: PLoS One. 2013 Feb 27;8(2):e57291. doi: 10.1371/journal.pone.0057291 (PMC3584116; doi:10.1371/journal.pone.0057291)

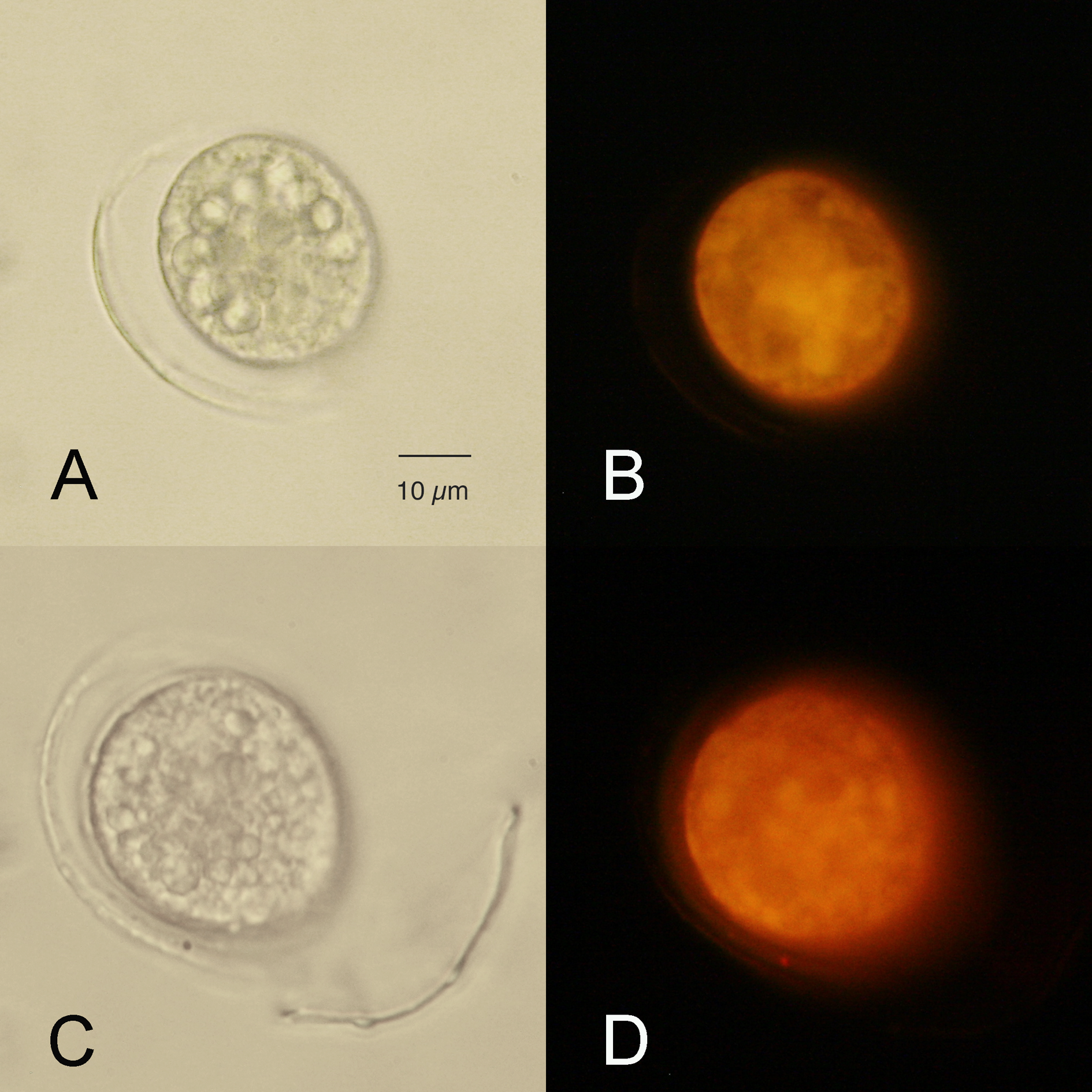

Supplement: Figure S1 — Ostreopsis cf. ovata depigmented cells stained with Nile Red. Cells were fixed with 2% paraformaldehyde (5 min at RT), depigmented upon washing 3 times with an 1∶1 acetone:hexane solution for 5 min and stained with Nile Red. Bright field microscopy (A,C); epifluorescence microscopy (B,D). Cells show a weak red and yellow orange fluorescence. Strongly yellow fluorescing lipid droplets are no more visible. (TIF) [file pone.0057291.s001.tif]

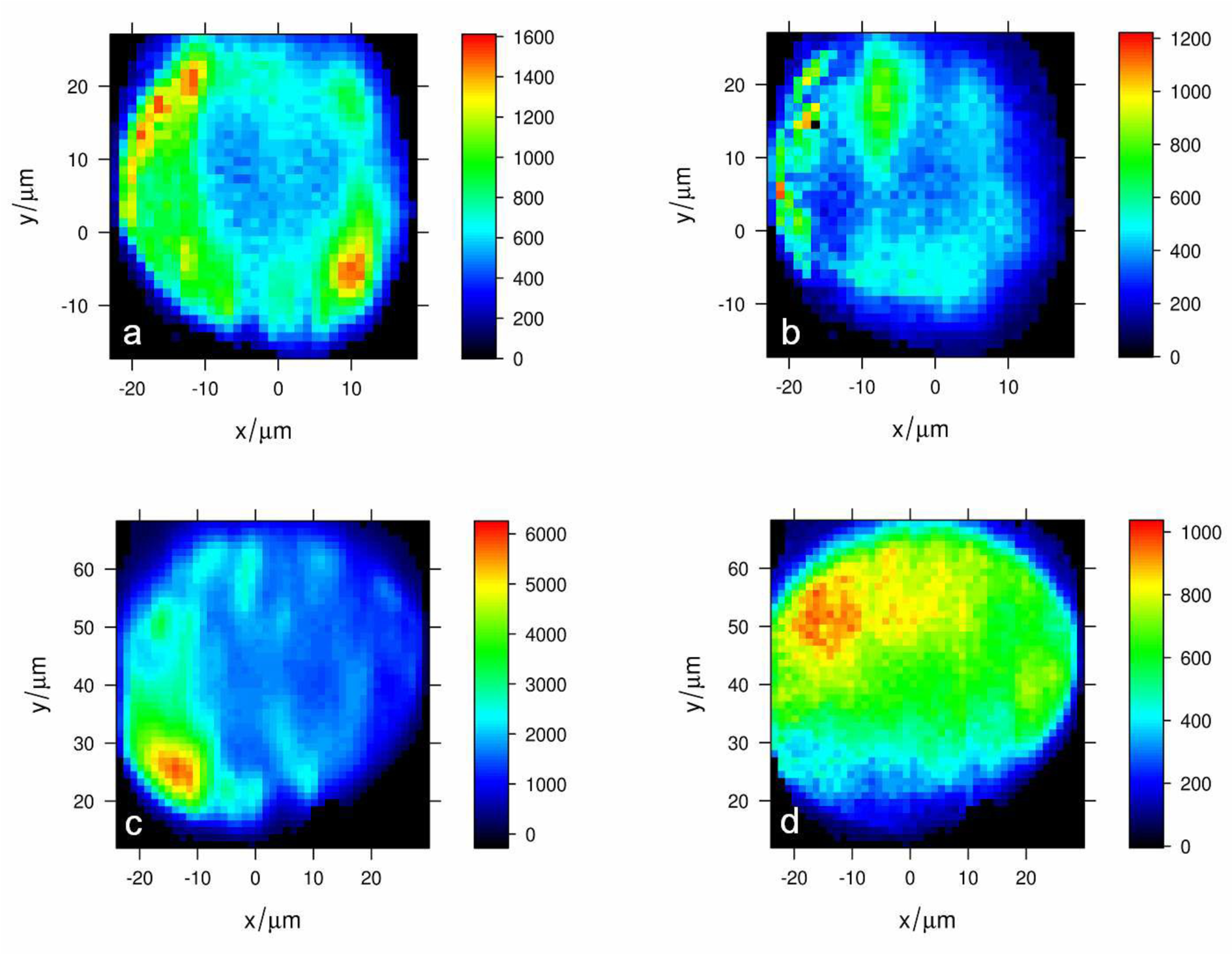

Supplement: Figure S2 — Raman maps depicting poly-unsaturated lipids and starch concentration of fixed and depigmented O. cf. ovata cells in the stationary and senescence phases. Raman maps depicting poly-unsaturated lipid concentration based on the un-normalized intensity at 1559 cm−1 of a fixed and depigmented O. cf. ovata cell in the stationary (a) and in the senescence (c) phases; Raman maps depicting starch concentration based on the un-normalized intensity at 941 cm−1 of a cell in the stationary(b) and in the senescence (d) phases. (TIF) [file pone.0057291.s002.tif]
